# First Reported Use of Recombinant Parathyroid Hormone in Kenny–Caffey Syndrome Type 2: A Case Report and Literature Review

**DOI:** 10.3390/diseases14030091

**Published:** 2026-03-03

**Authors:** Maja Djordjevic Milosevic, Anita Skakic, Marina Andjelkovic, Angelica Maria Delgado-Vega, Håkan Thonberg, Kristel Klaassen, Jovana Komazec, Bozica Kecman, Nikola Jocic, Erik Björck, Anna Lindstrand, Maja Stojiljkovic

**Affiliations:** 1 Institute for Mother and Child Healthcare of Serbia “Dr Vukan Cupic”, 11070 Belgrade, Serbia; mayamarko@gmail.com (M.D.M.);; 2 Faculty of Medicine, University of Belgrade, 11000 Belgrade, Serbia; 3 Institute of Molecular Genetics and Genetic Engineering, University of Belgrade, 11042 Belgrade, Serbia; anita.skakic@imgge.bg.ac.rs (A.S.); marina.andjelkovic@imgge.bg.ac.rs (M.A.); kristel.klaassen@imgge.bg.ac.rs (K.K.); jovana.komazec@imgge.bg.ac.rs (J.K.); nikola.jocic@imgge.bg.ac.rs (N.J.); 4 Department of Clinical Genetics and Genomics, Karolinska University Hospital, 17164 Stockholm, Sweden; angelica.delgado.vega@ki.se (A.M.D.-V.); hakan.thonberg@ki.se (H.T.); erik.bjorck@ki.se (E.B.); anna.lindstrand@ki.se (A.L.); 5 Department of Molecular Medicine and Surgery, Karolinska Institute, 17176 Stockholm, Sweden

**Keywords:** hypoparathyroidism, treatment, parathyroid hormone therapy, Kenny–Caffey syndrome type 2, *FAM111A* gene

## Abstract

**Background/Objectives**: Hypoparathyroidism (HPT) is a disorder caused by the insufficient production of parathyroid hormone (PTH). Its main features include decreased serum calcium, increased serum phosphorus, and abnormal bone modeling. In children, HPT is most commonly due to genetic disorders. Among rare genetic syndromes that can include HPT in their clinical spectrum is Kenny–Caffey syndrome (KCS) type 2. Conventional therapy for HPT primarily consists of oral calcium and active vitamin D metabolites. The major limitation of conventional therapy is hypercalciuria with an increased risk of nephrocalcinosis. However, a subset of patients fails to achieve the desired therapeutic response to conventional treatment; the reasons for this remain incompletely understood in some cases. The failure to achieve therapeutic targets and persistent hypercalciuria are the main indications for considering therapy with recombinant human parathyroid hormone (rhPTH). **Methods:** In addition to the review of the literature on rhPTH use in pediatric hypoparathyroidism, the first application of rhPTH in the treatment of genetically caused HPT in a child with Kenny–Caffey syndrome type 2 (KCS2) was described. **Results:** In this paper, we present a two-month-old infant who received rhPTH for 14 months. A heterozygous *de novo* p.Ser541Pro variant in the *FAM111A* gene was identified through whole-genome sequencing, indicating a diagnosis of KCS2. A biological mechanism linking FAM111A protein function with a more profound disruption of parathyroid development or function was proposed, suggesting that rhPTH therapy may be particularly beneficial in KCS2 cases. **Conclusions:** This is the first reported use of rhPTH in a child in Serbia and the first reported use in KCS type 2. By reviewing the literature, we analyzed the conditions in which rhPTH has been used, dosing approaches and durations, requirements for concomitant conventional therapy during rhPTH treatment, and the effects of rhPTH on calciuria. We provide an overview of rhPTH use in children. Additionally, based on the pathogenic genetic variant responsible for KCS2 in our patient, we propose possible etiologic explanations. This work aims to encourage a consideration of rhPTH use in children following its official approval.

## 1. Introduction

Hypoparathyroidism (HPT) is a disorder due to the insufficient production of parathyroid hormone (PTH). The main characteristics of hypoparathyroidism include a decreased calcium concentration, increased phosphorus concentration, and abnormal bone modeling. The causes of hypoparathyroidism are varied [1,2]. Unlike in adults, where the most common cause of HPT is surgery [3], in children, it is usually genetically determined [1]. The genetic causes of hypoparathyroidism can be divided into several groups [1,2]: disorders of parathyroid gland development, disorders of parathyroid hormone secretion, damage to parathyroid glands and hypomagnesemia syndromes. The group of disorders associated with the abnormal development of the parathyroid glands include syndromes such as: Di George syndrome (OMIM #188400), Hypoparathyroidism–Sensorineural Deafness–Renal Dysplasia (HDR) syndrome (OMIM #146255), Hypoparathyroidism–Retardation–Dysmorphism syndrome (HRDS/Sanjad-Sakati) (OMIM #241410), Kenny–Caffey syndrome (KCS) types 1 (OMIM #244460) and 2 (OMIM #127000) and some Mitochondrial diseases (like MELAS syndrome (OMIM #540000), Pearson Marrow–Pancreas syndrome (OMIM #557000), and Kearns–Sayre Syndrome (OMIM #530000)). The most common genetic disorder of PTH secretion results from pathogenic variants in the calcium-sensing receptor (CaSR). It belongs to the group of disorders characterized by isolated hypoparathyroidism and is also referred to as autosomal dominant hypocalcemia type 1 (ADH1) (OMIM #601198). Autoimmune Polyglandular Syndrome type 1 (APS-1) (OMIM #240300) is classified among disorders in which hypoparathyroidism arises from the genetically determined autoimmune-mediated destruction of the parathyroid glands. In addition, diverse genetic causes of hypomagnesemia may lead to impaired parathyroid hormone secretion. The clinical manifestations of hypoparathyroidism are primarily driven by hypocalcemia and exert widespread effects on multiple tissues and organ systems, notably the central nervous system, skeletal muscles, cardiovascular system, and kidneys [1,4]. Symptoms may include numbness and tingling in the extremities and perioral region, muscle cramps, and fatigue. However, they can lead to medical emergencies such as seizures, severe irregularities in normal heartbeat, laryngospasm, or bronchospasm. In the pediatric population, symptoms can occur already in the neonatal age but also later during childhood and adolescence [1,3]. Symptoms of hypocalcemia in children frequently emerge during rapid growth and puberty, due to increased skeletal calcium demand and the hormonal stimulation of bone mineralization [5] and infections, inflammatory stress, the alkalosis-induced reduction of ionized calcium, decreased intake, and increased metabolic demands [6].

The first-line treatment of hypoparathyroidism generally involves conventional therapy, which involves administering active forms of vitamin D and calcium salts orally. In cases of tetany and convulsions, calcium salts are administered intravenously [1,2]. There are many literature reports regarding the successful use of recombinant human parathyroid hormone (rhPTH) in patients of different ages with hypoparathyroidism [7,8,9,10,11,12,13,14,15,16,17,18,19,20,21,22,23,24,25,26,27,28,29,30,31,32]. However, there is still no official approval for the chronic use of rhPTH in children [1,2,3]. Thus, the use of rhPTH in children for the treatment of hypoparathyroidism is currently considered “off-label”. Nevertheless, published studies have reported its use in cases refractory to conventional therapy, predominantly in children with genetically determined hypoparathyroidism.

We present the case of a male neonate with hypoparathyroidism (HPT) occurring within the clinical spectrum of Kenny–Caffey syndrome type 2 (KCS type 2). The patient developed hypocalcemia that was refractory to conventional therapy. He was subsequently treated with recombinant human parathyroid hormone (rhPTH) and demonstrated an excellent clinical and biochemical response. To the best of our knowledge, this represents the first administration of rhPTH in a child in Serbia. In addition, we provide a review of the literature addressing the use of rhPTH in pediatric patients with genetically determined HPT of various etiologies. To our knowledge, this is also the first published report describing the use of rhPTH in an individual with KCS type 2.

## 2. Case Report

A male neonate was born via cesarean section at 36 weeks due to prenatal growth restriction, with a birth weight of 2350 g (<P10) and a length of 46 cm (>P10). After birth, he underwent phototherapy for indirect hyperbilirubinemia. No dysmorphic features were observed, and his family history was unremarkable. At the 16th day of life, he was admitted to the Neonatal Intensive Care Unit (NICU) due to recurrent seizures persisting for more than two days. Immediately upon admission, intravenous phenobarbital was administered at a loading dose of 10 mg/kg for termination of the ongoing seizure, followed by maintenance oral phenobarbital at 3 mg/kg/day. Continuous cardiorespiratory monitoring was initiated, including heart rate, respiratory rate, and continuous electrocardiographic (ECG) monitoring. The initial laboratory evaluation revealed: total serum calcium level of 1.2 mmol/L (reference range, 2.05–2.74 mmol/L), serum phosphorus level of 4.35 mmol/L (age-specific reference range, 1.15–2.15 mmol/L), and serum magnesium level of 0.56 mmol/L (reference range, 0.7–1.05 mmol/L). During evaluation, the neonate experienced another convulsive episode. A slow intravenous bolus of 10% calcium gluconate was administered at a dose of 1 mL/kg (2.5 mL of 10% calcium gluconate diluted with 2.5 mL of 0.9% sodium chloride), under continuous ECG and vital sign monitoring. After seizure cessation, intravenous calcium supplementation was continued as a continuous infusion of 10% calcium gluconate at 2 mL/kg/24 h via a syringe pump. The infusion solution consisted of a total volume of 50 mL containing 5% dextrose and 0.9% sodium chloride in a 6.5:1 ratio. The biochemical profile (hypocalcemia with hyperphosphatemia) raised the suspicion of hypoparathyroidism, which was confirmed on the second day of hospitalization by a markedly decreased parathyroid hormone (PTH) level < 3.0 pg/mL (reference range for neonate, 10–60 pg/mL). On the same day, Dedyiol^®^ (alpha calcidiol) was initiated at 1 drop twice daily and gradually titrated to 5 drops twice daily (total 10 drops per day, equivalent to 0.375 µg of alpha calcidiol). The liquid formulation was selected because of its suitability for administration in neonates and young infants, as well as for more precise dose titration. Oral magnesium oxide was also introduced at a dose of 25 mg twice daily in powder form. On the third day of hospitalization, in addition to parenteral calcium, oral calcium carbonate was initiated at a dose of 1 g/kg/24 h (body weight at that time 2.5 kg), administered as a powder mixed with milk formula. During the subsequent ten days, due to persistently low serum calcium concentrations, the dose of parenteral intravenous 10% calcium gluconate was increased to 4 mL/kg/24 h (corresponding to 0.8 mmol/kg/24 h or 36 mg/kg/24 h of elemental calcium). The remainder of the therapy consisted of alpha calcidiol 0.375 µg/day, oral calcium carbonate 1 g/kg/24 h, magnesium oxide 25 mg twice daily, and oral phenobarbital 3 mg/kg/day. The patient remained seizure-free, and total serum calcium levels were maintained above 2.0 mmol/L. Between the fifteenth and twentieth day of hospitalization, intravenous calcium gluconate was gradually tapered and discontinued. This resulted in a decline of total serum calcium to 1.25 mmol/L and the recurrence of convulsive seizures. The seizure was terminated with an intravenous bolus of 10% calcium gluconate, and continuous intravenous administration was reintroduced at 4 mL/kg/24 h. Due to the need for prolonged high-dose parenteral calcium administration, a central venous catheter was placed. The continuous monitoring of vital signs and ECG was maintained. Calcitriol (Rocaltrol^®^) was added to the therapeutic regimen, initially at 0.25 µg once daily and subsequently increased after several days to 0.25 µg three times daily. Parenteral calcium supplementation was continued, while the remainder of therapy remained unchanged. On the fortieth day of life (23rd hospital day), the clinical course was complicated by Pseudomonas sepsis, for which intravenous meropenem therapy was administered for two weeks. During this period, the patient continuously received 10% calcium gluconate intravenously at 4 mL/kg/24 h. Total serum calcium levels did not decrease below 1.72 mmol/L, and no further seizures occurred.

**Figure 1:**
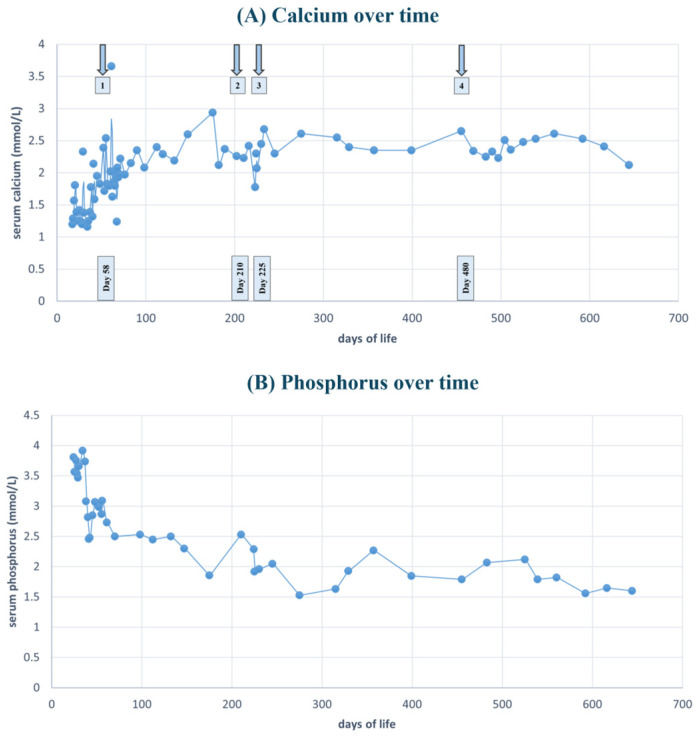
(**A**) illustrates the trend in serum calcium concentration before and during rhPTH therapy and follow-up period on conventional therapy alone. Arrow 1 indicates the day of rhPTH initiation; arrow 2 marks the day of rhPTH discontinuation; arrow 3 denotes the day of rhPTH reintroduction; and arrow 4 indicates the day of final discontinuation of rhPTH therapy. (**B**) illustrates the trend in serum phosphorus before and during rhPTH therapy and follow-up period on conventional therapy alone.

Considering the persistent hypocalcemia, the development of sepsis, and the risks associated with prolonged central venous catheterization (including pleural effusion, pericardial effusion, thrombosis, and thromboembolic complications) [33], the medical team decided to initiate treatment with recombinant human parathyroid hormone (rhPTH) teriparatide, the only available rhPTH preparation in Serbia. Therapy was initiated on the 58th day of life at a dose of 0.54 µg/kg/24 h, administered subcutaneously and divided into two daily doses, following approval by the Ethics Committee of our University Hospital (Approval No. 08/79, 3 October 2023), in accordance with all applicable legal and regulatory standards. During the first days of therapy, total serum calcium levels were monitored twice daily. The patient remained seizure-free; however, calcium concentrations fluctuated, with both lower and higher values recorded. On the third day after initiation of teriparatide, total serum calcium increased to 3.66 mmol/L, prompting the rapid discontinuation of intravenous 10% calcium gluconate. During the following ten days, the patient remained seizure-free. At one point, the total serum calcium decreased to 1.63 mmol/L, leading to a temporary increase in the teriparatide dose to 0.6 µg/kg/24 h. Alpha calcidiol was gradually discontinued, and oral calcium carbonate intake was reduced. At discharge on the 70th day of life, the infant weighed 4 kg. Total serum calcium was 2.2 mmol/L. Ongoing therapy consisted of subcutaneous teriparatide 0.55 µg/kg/24 h (total 2.2 µg/24 h), divided into two daily doses, phenobarbital 3 mg/kg/day, calcitriol 0.25 µg three times daily, calcium carbonate 1 g/day, and magnesium oxide 25 mg twice daily. During follow-up, phenobarbital and oral calcium carbonate were discontinued within the first two months. Calcitriol was reduced to 0.25 µg twice daily, magnesium oxide remained at 25 mg twice daily, and teriparatide was continued at the same total daily dose of 2.2 µg/24 h subcutaneously. The urinary calcium-to-creatinine ratio (mmol/mmol) was around 0.5. At six months of age, the patient’s serum calcium was slightly above the upper reference limit (2.78 mmol/L), while the urinary calcium-to-creatinine ratio was 1 mmol/mmol [34]. Consequently, the teriparatide dose was reduced by half, to 1.1 µg per 24 h. At seven months, an attempt was made to discontinue teriparatide; however, after two weeks, hypocalcemia recurred (1.91 mmol/L), necessitating the reintroduction of teriparatide at the original daily dose of 2.2 µg per 24 h. During the subsequent seven months, no episodes of hypocalcemia were observed, and the urinary calcium-to-creatinine ratio remained ≤0.5 mmol/mmol. Teriparatide was successfully discontinued after fourteen months of therapy without the recurrence of hypocalcemia. The calcitriol dose was reduced to 0.25 µg per 24 h after several measurements showed a total serum calcium of 2.48 mmol/L and an elevated urinary calcium-to-creatinine ratio of 0.9 mmol/mmol. Magnesium oxide therapy was unchanged. In the following months, the child remained normocalcemic, with urinary calcium excretion within normal limits. Table 1 summarizes the pharmacologic treatment regimen before, during, and eight months following the discontinuation of rhPTH therapy. Figure 1A illustrates the total serum calcium levels from hospital admission through to day 600 of life. Arrows indicate the initiation of teriparatide therapy, the first attempt at treatment discontinuation, and the final cessation of rhPTH therapy. Figure 1B depicts the time course required for the gradual normalization of serum phosphorus levels.

At two years of age, our patient remains on conventional therapy with calcitriol 0.25 µg once daily and magnesium oxide 25 mg twice daily. Follow-up evaluations are performed every three months and include a clinical examination and measurement of serum calcium and phosphorus levels, which have remained within the reference range. The PTH level is below the lower limit of normal (<6 pg/mL), and the urinary calcium-to-creatinine ratio (mmol/mmol) is ≤0.5. Renal ultrasonography shows no evidence of nephrocalcinosis. He currently presents with short stature, significantly below −2 standard deviations for age and sex. The anterior fontanelle closed within the expected timeframe (at 15 months of age). No dysmorphic features have been observed, including frontal bossing, microphthalmia, micrognathia, or other ocular anomalies. To date, all primary (deciduous) teeth have erupted; however, they are discolored and exhibit punctate yellowish deposits. Although short stature is recognized as part of the clinical spectrum of Kenny–Caffey syndrome type 2, further evaluation is planned in light of the child’s growth impairment. This will include a comprehensive hormonal assessment, X-ray of the left hand and wrist to assess bone age and radiological studies aimed at evaluating cortical thickening and medullary stenosis of the tubular long bones, in consideration of potential growth hormone therapy.

Considering the neonatal onset of hypoparathyroidism and the inadequate response to conventional therapy, early evaluation for a genetic etiology was warranted during the neonatal period. Chromosomal microarray analysis was performed and yielded normal results, thereby largely excluding Di George syndrome.

**Figure 2:**
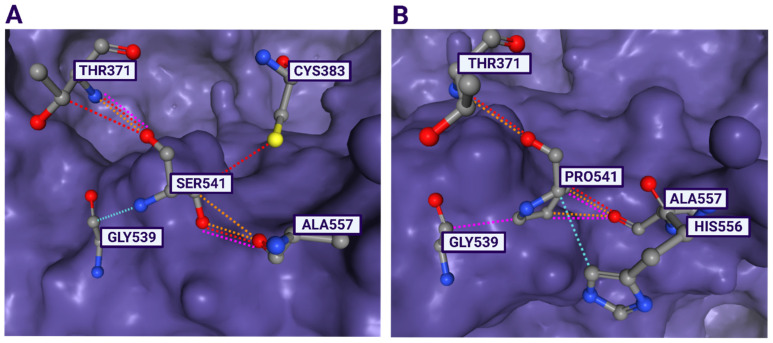
The three-dimensional molecular model of the FAM111A protein. The close-up view of the region harboring the identified variant (p.Ser541) (**A**) and wild type (p.Pro541) (**B**) was analyzed using Dynamut2 with the PDB code 8S9L. Hydrogen bonds are shown in red dotted lines, Van der Waals interactions are shown in blue dotted lines, polar interactions are shown in orange dotted lines, and clashes are shown in pink dotted lines. A change at p.Ser541 affects the catalytic triad composed of Asp439-His385-Ser541, while Dynamut2 additionally predicts altered interactions with residues Gly539, Thr371 and Ala557, the abolishment of a hydrogen bond with Cys383, and a novel interaction with His 556 in case of p.Pro541. Created in BioRender. Komazec, J. (2026), https://BioRender.com/sdvfu0z (accessed on 19 December 2025).

Short-read whole-genome sequencing (SR-WGS) was performed in the second year of a patient’s life to elucidate the underlying molecular genetic base. SR-WGS identified a heterozygous c.1621T > C, p.(Ser541Pro) variant in the *FAM111A* gene (NM_001312909.2), as the patient’s clinical presentation closely correlated with previously described cases of Kenny–Caffey syndrome type 2 [35,36,37]. Additionally, whole-exome sequencing of both biological parents confirmed that the variant arose *de novo* in the patient. Furthermore, we applied ACMG/AMP’s guidance for the interpretation of sequence variants [38] in order to precisely assess the pathogenicity of the p.Ser541Pro variant. This is a rare variant, previously not found in the databases of healthy people, such as gnomAD exomes and gnomAD genomes. The 541 amino acid residue is highly conserved across species, implying its important role in the protein. Furthermore, different computational predictor tools unambiguously predicted that a serine-to-proline substitution at the 541 amino acid residue must have a deleterious effect upon the structure of the FAM111A protein (e.g., REVEL score 0.831, ClinPred score 0.9988 and MetaRNN score 0.8837, etc.). The three-dimensional molecular model of the FAM111A protein clearly shows that a change at p.Ser541 affects the catalytic triad composed of Asp439-His385-Ser541 (Figure 2). In addition to p.Ser541Pro, another pathogenic missense change at the same amino acid residue, p.Ser541Tyr, had been previously reported [37]. Moreover, recent structural studies of the FAM111A protein have highlighted the crucial functional importance of the Ser541 residue, which forms part of the catalytic machinery of the C-terminal trypsin-like protease domain [39]. Based on all presented evidences, the variants’ rarity in a healthy population (PM2), its phenotypic specificity (PP4 and PP5), the *de novo* occurrence with both maternity and paternity confirmed (PS2), computational evidences (PP3), and the functional relevance of the affected residue (PS3 and PM5), the variant was classified as pathogenic according to ACMG/AMP criteria [38]. Therefore, the finding of the p.Ser541Pro variant in the *FAM111A* gene establishes a definitive molecular diagnosis of Kenny–Caffey syndrome type 2 in this patient and provides the genetic context for the first reported use of recombinant parathyroid hormone therapy in this condition.

## 3. Discussion

The etiology of hypoparathyroidism in childhood is heterogeneous; however, in the majority of patients, the underlying cause is genetic. Hypoparathyroidism most commonly occurs as part of the clinical spectrum of various genetic disorders, although it may also present as an isolated manifestation of pathogenic genetic variants [1,2].

Comprehensive genetic analyses such as WES and WGS represent powerful tools to unravel overlapping or complex phenotypes, precisely pinpointing to disease-causing variants and shedding light on the molecular basis of heterogeneous clinical manifestations [40,41,42,43]. In the present case, the SR-WGS identified a pathogenic p.Ser541Pro variant in the *FAM111A* gene which causes Kenny–Caffey Syndrome type 2 (KCS2). KCS2 is a very rare genetic disorder, inherited in an autosomal dominant manner and characterized by primary hypoparathyroidism (which may be transient or remittent), medullary stenosis of the long bones, proportionate short stature, ocular abnormalities, and a delayed closure of the anterior fontanelle [44]. Some patients may also exhibit clinical features that include the calcification of the basal ganglia, anemia, the dysfunction of T-cells, minor genitourinary anomalies, microorchidism or infertility in affected males, small hands and feet, and orofacial findings (elfin facies with hypoplasia of the mid-face, telecanthus, small palpebral fissures, small pinched upturned nose and a small chin [36]; Eustachian tube dysfunction may appear later [37]. KCS2 was first described in the literature in 1966 [45]. Since the first reported case, only 61 cases with an identified *FAM111A* pathogenic variant have been reported globally [42]. Parathyroid gland dysfunction in KCS type 2 syndrome results from an abnormal development of the parathyroid glands, which is genetically determined [1].

A literature search was performed in the PubMed database using the following search terms: “children,” “hypoparathyroidism,” and “treatment with rhPTH.” No restrictions were applied, including the duration of publication years. In total, 156 articles were retrieved. When multiple publications from the same author group provided updated recommendations on diagnosis and treatment, only the most recent publication was included. Of the 156 retrieved articles, those addressing lactation and breastfeeding were excluded from the analysis. We did not analyze patients in whom hypoparathyroidism was a consequence of surgical intervention or radiation therapy; however, their number is presented in Table 2. Importantly, in 2019, Dayal et al. [8] provided a comprehensive and detailed analysis of different types of hypoparathyroidism in children treated with rhPTH. Eight publications (published after 2019) reported the use of rhPTH in pediatric patients with HPT of various etiologies, encompassing a total of 23 children [23,24,25,26,27,28,29,30].

For decades, hypoparathyroidism was considered the only classical hormone deficiency disorder not treated with hormone replacement therapy [2,46,47]. Conventional therapy for HPT involves the administration of active forms of vitamin D and calcium, achieving normocalcemia in most patients. However, pronounced hypercalciuria and resistance to conventional therapy have been the primary reasons for considering substitution therapy [3,8,9]. Hypercalciuria is an integral part of the clinical picture in individuals with HPT, and therapy with active forms of vitamin D and calcium increases hypercalciuria, thereby raising the risk of nephrocalcinosis. Consequently, there are challenges associated with conventional therapy. Today, the treatment of HPT in adults also includes the use of rhPTH, whereas this therapy in children has not yet been approved by the Food and Drug Administration (FDA) and European Medicines Agency (EMA), although it is used “off-label”. The following forms of rhPTH have been used in the treatment of HPT to date: rhPTH (1-34) (teriparatide), rhPTH (1-84), and Palopegteriparatide [46,48].

Recombinant human parathyroid hormone rhPTH (1-84) was approved in 2015 for the treatment of adults with chronic hypoparathyroidism inadequately controlled with calcium conventional therapy. It is administered subcutaneously once daily. However, the production of rhPTH (1-84) was discontinued in the USA in 2019 and in Europe in 2024 [48]. Data on the use of teriparatide in the treatment of hypoparathyroidism date back to 1996 [49]. Teriparatide was FDA-approved in 2002 for treating osteoporosis in postmenopausal women and male osteoporosis secondary to hypogonadism [50]. Although it has not been officially approved for use in children, there are reports of successful “off-label” use of the drug, primarily short-term, in the treatment of resistant hypoparathyroidism in children unresponsive to conventional therapy. Recommended daily doses for children of all ages for rhPTH (1-34) range from 0.34 to 1.4 μg/kg/day, and the preparation has also been administered continuously via pumps (for injection) [2]. Palopegteriparatide is a preparation that provides a continuous release of rhPTH (1-34) and maintains stable PTH levels in the physiological range for 24 h per day. In 2024, it was officially approved by the FDA and EMA for the treatment of hypoparathyroidism in adults [48].

The advantages of rhPTH therapy include reduced urinary calcium excretion, lower requirements for calcium and active vitamin D, decreased risk of ectopic calcification, improved bone remodeling, and enhanced quality of life [6,51]. Concerns about long-term use in children arise from reported bone tumors in rat models [52,53]. Some studies question whether toxicity data from rat models can be directly applied to humans due to the significant differences in skeletal physiology between rodents and primates, the long treatment duration in rats (almost all their lifespan), and the use of very high, supra-physiological doses. Additionally, post-marketing surveillance data from fifteen years of experience with rhPTH (1-34) and over ten years with rhPTH (1-84) did not find the aforementioned association. Ultimately, its routine use in children is still not recommended due to a lack of documentation of its advantages compared to conventional therapy [8,14]. However, the black box warning regarding the risk of osteosarcoma associated with teriparatide has been removed, and there is a strong indication that rhPTH will soon be approved for use in children [53,54].

In 2019, Dayal et al. [8] provided an overview of published studies regarding the use of rhPTH in children with various causes of hypoparathyroidism [9,10,11,12,13,14,15,16,17,18,19,20,21,22,23]. The estimated number of patients reported in the literature at that time was around 70 [8]. The exact number of pediatric patients treated with rhPTH was difficult to determine, as reports from the United States by Winer et al. [14,18,19] appear to include the same patients analyzed repeatedly from different perspectives during rhPTH therapy. In this study, all children were treated with rhPTH (1-34) [8]. Among the 15 reported experiences in this study, continuous administration via an infusion pump was used in three reports [17,21,25], whereas the remaining studies described single (a varying number of times) subcutaneous injections [10,11,12,13,15,16,17,18,20,21,22,23,24]. The majority of these children with HPT were diagnosed with Autoimmune Polyglandular Syndrome type 1 (APS type 1) [14,15,18,19,20,22,23,24] and calcium-sensing receptor (CaSR)-related HPT [9,11,13,14,18,21,24]. Cases in which the underlying cause of hypoparathyroidism remained unidentified have also been reported [12,15,18,23]. Individual cases of rhPTH application include children with DiGeorge syndrome [20] and very rare syndromes such as Sanjad–Sakati syndrome [17] and hypoparathyroidism with deafness and renal dysplasia (HDR) [20]. The children from the Dayal et al. study [8] were of different ages, and the duration of rhPTH therapy varied widely, ranging from a single subcutaneous injection in a newborn [10] to a long-term treatment lasting 13.5 years [15]. Dayal et al. [8] concluded that rhPTH therapy in children with hypoparathyroidism has not been associated with significant adverse outcomes, including skeletal malignancies. Nevertheless, they underscored the need for additional long-term studies to further clarify the safety profile of prolonged rhPTH administration, particularly with respect to hypercalciuria, nephrocalcinosis, nephrolithiasis, renal impairment, and ectopic calcifications.

Since 2019, more than 20 cases on the use of rhPTH in children have been identified in the literature (Table 2) [25,26,27,28,29,30,31,32]. The advancement and availability of genetic testing have resulted in a decreased number of children with idiopathic hypoparathyroidism, as indicated in this study (Table 2). The majority of patients presented in Table 2 had diagnoses of autoimmune APS type 1 (n = 7) [26,27,28] and CaSR-related hypoparathyroidism (n = 7) [25,26,32], followed by patients with Di George syndrome (n = 4) [26,27,29]. Individual case reports were also described in patients with HDR syndrome [27], Jacobsen syndrome [30], Sanjad–Sakati syndrome [31] and our patient with Kenny–Caffey syndrome type 2. No allergic responses were observed [25,26,27,28,29,30,31,32]. Two reports that we presented in Table 2 have described the use of rhPTH (1-84) for the treatment of hypoparathyroidism [25,28], while other pediatric reports are related to treatment with teriparatide (rhPTH 1-34) [26,27,29,30,31,32]. The doses of rhPTH (1-34) administered in the patients presented in Table 2 ranged from 0.31 µg/kg/24 h to a total daily dose of 25 µg/24 h, whereas the doses of rhPTH (1-84) ranged from 3.2 µg/kg/24 h to a total daily dose of 75 µg/24 h. Continuous pump therapy was used only briefly in two children [26,31].

In the majority of the patients presented in Table 2 [25,26,27,29,32], rhPTH therapy was associated with a reduction in the doses of calcium and active vitamin D, and in some cases these treatments were temporarily discontinued. It may be concluded that the decision to continue conventional therapy should be individualized and based on the careful monitoring of the clinical course and laboratory parameters. Calciuria decreased, however, in all presented patients; nephrocalcinosis either worsened or newly developed (Table 2), warranting further discussion. A possible explanation for the occurrence of nephrocalcinosis despite the reduced calciuria was proposed by Tuli et al. [27]. They suggested that calcium may have accumulated in the urinary system during prior conventional therapy, often compounded by the underlying disease itself, thereby substantially increasing the risk of nephrocalcinosis, which could not be fully mitigated by transitioning to rhPTH therapy [31].

Considerations related to disease etiology and the indications for rhPTH administration should be tailored to the underlying cause of hypoparathyroidism. In patients with Autoimmune Polyglandular Syndrome type 1 (APS type 1), the resistance to conventional therapy is likely due to malabsorption [26,27,28]. The majority of patients treated with rhPTH so far have been children with pathogenic variants in the CaSR gene, representing the most common cause of isolated hypoparathyroidism in children (Table 2). Patients with CaSR pathogenic variants may benefit the most from rhPTH therapy compared to conventional treatment because it reduces hypercalciuria (which is otherwise pronounced in patients with CaSR pathogenic variants and is exacerbated by the use of the active form of vitamin D), thereby lowering the risk of nephrocalcinosis [8,18,26]. Man Tong made an interesting observation about the synergistic effects of teriparatide and native vitamin D. He observed that the administration of native vitamin D and calcium significantly reduced the required dose of rhPTH. The reason for initiating native vitamin D therapy was a low serum 25-hydroxyvitamin D (25OHD) level [32].

Bernardor et al. reported metabolic crises in one child during rhPTH therapy, which were attributed to the development of both hypercalcemia and hypocalcemia and were associated with the mode of drug administration via a continuous infusion pump. This issue was resolved by switching to twice-daily subcutaneous injections [26]. However, Bali et al. also reported the development of hypercalcemia after a short course of therapy, and the transition from intermittent dosing to continuous rhPTH administration (the same total 24 h dose) [31]. Based on the reports published to date in which a continuous rhPTH infusion via pump was employed, both the advantages and disadvantages of this mode of administration have been described. The potential benefits include the provision of more physiological conditions for calcium homeostasis [19] and lower urinary calcium excretion compared with single-dose administration [27]. Conversely, the concerns include possible technical difficulties, the burden of pump wear in pediatric patients [28], and the risk of hypercalcemia [31].

Our patient’s treatment with teriparatide began at the age of two months, although the first symptoms appeared during the neonatal period. The duration of teriparatide therapy was fourteen months. The youngest patient described in the literature who received long-term teriparatide therapy for six months was a 4-month-old preterm child with DiGeorge syndrome [29]. The use of teriparatide even at neonatal age is documented in the literature, but only short-term use with a single dose has been reported. Newfield described a 17-day-old Hispanic boy with resistant hypoparathyroidism who received 5 µg of teriparatide subcutaneously in one dose nearly two decades ago. He pointed out that the short-term use of teriparatide should be further evaluated for its advantage in raising calcium more rapidly and safely than other methods [12].

In our patient with KCS type 2 syndrome, severe refractory hypocalcemia was observed in the neonatal period despite conventional oral therapy for HPT treatment. The lack of a therapeutic response could be attributed to transient malabsorption and/or exocrine pancreatic insufficiency, although this was not confirmed; normal stool characteristics and appropriate weight gain argued against these possibilities. On the other hand, it is noteworthy that normocalcemia was achieved only with parenteral calcium supplementation and subsequently with rhPTH s.c. therapy. In addition, the immaturity of various enzyme systems and receptor pathways in the neonatal period should be considered as a potential contributing factor. Another possible explanation could be reduced 25-hydroxyvitamin D (25OHD) levels in the child, secondary either to maternal deficiency or to congenital disorders of vitamin D metabolism, although this was not confirmed. For all the above reasons, it is reasonable to consider a potential association between resistant hypocalcemia and various genetically determined forms of hypoparathyroidism.

At present, there is no direct experimental evidence linking the *FAM111A* gene, affected in the present patient, to hypoparathyroidism. FAM111A (FAM111 trypsin-like peptidase A) is a serine protease involved in multiple cellular processes including antiviral defense and DNA replication [39].

Structural and functional studies have demonstrated that the C-terminal domain of FAM111A contains a conserved catalytic triad typical of trypsin-like peptidases (His385–Asp439–Ser541), with Ser541 functionally identified as the catalytic serine by mutational analyses showing a loss of protease activity upon substitution with an amino acid other than serine [39,55,56]. The replacement of this crucial serine residue with the proline residue is therefore predicted to disrupt the local secondary structure and active-site geometry, especially as proline residues impose rigid conformational constraints on the peptide backbone and are known to perturb enzymatic active sites and protein–protein interactions [57].

Knowing that the protease activity of FAM111A needs to be tightly regulated for proper DNA replication and cellular homeostasis [39], the pathogenic variant p.(Ser541Pro) can be plausibly connected to hypoparathyroidism through replication-associated cellular stress in highly proliferative progenitor cell populations [55,58,59]. Namely, parathyroid gland size and its functional reserve appear to be largely determined during early embryogenesis through the tightly regulated expansion of a finite progenitor cell pool, with a limited capacity for postnatal regeneration, a principle supported by clinical and genetic studies of congenital hypoparathyroidism [58,60]. Consequently, developmental disturbances that impair progenitor proliferation, survival, or differentiation may result in lifelong hypoparathyroidism characterized by a residual but insufficient hormone production [61].

Therefore, we suggest that irregular FAM111A activity, which causes improper DNA replication and subsequently affects the proliferation of parathyroid progenitor cells, likely leads to insufficient gland development, compromising its postnatal function and subsequent hormone deficiency [55,59,60]. This proposed mechanism entails indirect effects on parathyroid development and progenitor cell viability rather than the direct modulation of parathyroid hormone synthesis or secretion in mature glandular tissue.

Taken together, this is a biologically coherent explanation for neonatal-onset hypocalcemia and hypoparathyroidism in the present patient, as well as in previously reported monozygotic twins harboring the same p.(Ser541Pro) variant [62].

Currently, there are no clinical or preclinical studies reporting the targeted therapeutic correction of FAM111A dysfunction. In this context, therapeutic repurposing strategies that target downstream cellular stress pathways rather than the primary genetic defect itself may represent a feasible and translatable approach. We and others have previously demonstrated that the modulation of endoplasmic reticulum stress, apoptosis, and cellular homeostasis using repurposed small molecules can partially rescue disease-relevant phenotypes in inherited metabolic disorders [63,64,65,66]. These studies highlight the potential of pathway-directed interventions to mitigate cellular vulnerability. Although such approaches remain speculative at present, they underscore the importance of exploring precision therapeutic strategies to improve the lives of patients with KCS2.

## 4. Conclusions

Conventional therapy achieves the normalization of serum calcium levels in the majority of children with hypoparathyroidism. However, treatment-refractory cases have been reported, particularly among patients with genetically determined parathyroid dysfunction who fail to respond adequately to standard therapy. The resistance to conventional treatment may be attributable to gastrointestinal dysfunction, abnormalities in vitamin D metabolism, or the genetic basis of the underlying disorder. Beyond providing an improved control of hypocalcemia, rhPTH therapy has also been associated with a reduced risk of hypercalciuria. The comprehensive reporting of efficacy and safety outcomes for rhPTH therapy in pediatric patients remains essential. To date, pediatric use of rhPTH has not been associated with significant adverse effects, apart from those related to dose adjustments, in contrast to the findings from preclinical studies in rats. These observations support the continuation of clinical investigations and may ultimately justify the routine use of rhPTH in selected pediatric populations. The reports of individual pediatric cases involving various genetically determined forms of hypoparathyroidism have been largely encouraging. The present study describes the first pediatric patient in Serbia to receive rhPTH replacement therapy and represents the first reported use of rhPTH in a child with Kenny–Caffey syndrome type 2.

Moreover, this study proposes a biological mechanism of action connecting the function of the FAM111A protein with a more profound disruption of parathyroid development or function, implying that rhPTH therapy might be especially beneficial in such cases. Besides Ser541 residue, our hypothesis may be extrapolated to other putative catalytic triad residues (His385 and Asp439), suggesting a more favorable response to rhPTH in comparison to conventional therapy. However, this hypothesis requires validation in larger patient cohorts and functional studies.

## Figures and Tables

**Table 1:** Overview of pharmacological therapy before, during, and after rhPTH treatment.

Medication	rhPTH Treatment (Teriparatide (2.2 µg/24 h)		
	Before	During (Interruption 15 Days)	After
	From 16 to 58 Days of Life (1.5 Month)	From 60 to 480 Days of Life (14 Months)	From 480 to 680 Days of Life (6 Months)
Calcium gluconate 10% i.v. continuous infusion	2 mL/kg/24 h for three days; maximum 4 mL/kg/24 h Discontinuation of continuous infusion was attempted twice	Abruptly discontinued on day 3 after initiation of rhPTH due to hypercalcemia	-
Calcium carbonate (oral)	1 g/kg	Discontinued after two months of teriparatide therapy	-
Dediyol^®^ (calcidiol) oral drops	0.375 µg/24 h	0.375 µg/24 h	-
Rocaltrol^®^ (calcitriol) soft capsules, oral	3 × 0.25 µg	2 × 0.25 µg	1 × 0.25 µg ongoing
Magnesium oxide (MgO) powder, oral	2 × 25 mg	2 × 25 mg	2 × 25 mg
Phenobarbital (oral)	3 mg/kg	Discontinued after two months of teriparatide therapy	-

Note: The bolus dose of 10% calcium gluconate administered for the management of seizures secondary to hypocalcemia is not included in this table, as it is described in detail in the main text.

**Table 2:** Review of rhPTH treatment in pediatric HPT.

Author, Year of Publication	The Underlying Disease	Number of the Patients	Age When rhPTH Started	Daly Dosage of rhPTH	Type of rhPTH and Dosing Regimen	Duration of rhPTH Therapy	Calciuria and Nephrocalcinosis
Tuli et al., 2020 [27]	APS type 1 Sy Di George 2 HDR 1	3 2 1	3.4–8.3 y 8.9–10.7 y 18.6 y	0.31–0.82 μg/kg/daily, maximum 25 μg/daily	rhPTH (1-34) s.c. twice daily	9.2 y	Reduction in calciuria in all children during rhPTH therapy; however, nephrocalcinosis developed in two patients
Hawkes et al., 2020 [25]	CaSR	2	9.5 y 9.5 y	50–75 μg/daily 25–50 μg/daily	rhPTH (1-84) s.c. once daily	18 mo	Reduction in calciuria in both children during rhPTH therapy
Laurer et al., 2021 [28]	APS type 1	1	5 y	0.32 μg/kg/ daily	rhPTH (1-84) s.c twice daily	4 mo	The upper limit of calciuria during rhPTH therapy was lower compared with conventional therapy, during which urinary calcium levels were above the normal range
Bernardor et al., 2021 [26]	APS type 1 CaSR Di George 1 Post-surgery Unknown	3 4 1 1 1	4.1–11.2 y 7.6–11.5 y 12.3 y 15.5 y 15.5 y	0.7–1.5 µg/kg/daily Max 20 µg twice daily	rhPTH (1-34) s.c twice daily	For at least one year of follow-up	Reduction in calciuria during rhPTH therapy; however, nephrocalcinosis worsened in five patients
Dayal 2021 [30]	Sy Jacobsen	1	7 y	0.55 µg/kg/daily (starting) 0.26 μg/kg g/kg/daily	rhPTH (1-34) s.c. twice daily	10 y	Reduction in hypercalciuria and absence of nephrocalcinosis during the last three years of rhPTH therapy
Neelan et al., 2022 [29]	Sy Di George	1	4 mo	0.7 μg/kg/daily	rhPTH (1-34) s.c. twice daily	6 mo	Nephrocalcinosis was neither present prior to nor observed after rhPTH therapy
Bali et al., 2024 [31]	Sy Sanjad Sakati	1	2 mo	1–1.5 μg/kg/daily	rhPTH (1-34) single dose s.c. (6 days) and continuous by pump (1 day)	A few days	The duration of rhPTH therapy was too short to assess trends in calciuria
Tong and Wong 2025 [32]	CaSR	1	16 mo	0.5 μg/kg/daily	rhPTH (1-34) s.c. twice daily	10 y	Early nephrocalcinosis was present but remained stable during rhPTH therapy
The case of our patient	Sy KC type 2	1	2 mo	0.55–0.60 μg/kg/daily	rhPTH (1-34) s.c. twice daily	14 mo	Calciuria decreased during rhPTH therapy, with no evidence of nephrocalcinosis

Legend: s.c.—subcutaneous application; HDR sy—hypoparathyroidism, deafness and renal dysplasia (kidney malformation); APS type 1-Autoimmune Polyendocrine Syndrome type 1; CaSR—calcium-sensing receptor; Sy KC type 2—Kenny–Caffey syndrome type 2.

## Data Availability

The original contributions presented in this study are included in the article. Further inquiries can be directed to the corresponding author.

## Abbreviations

The following abbreviations are used in this manuscript:

HPT	Hypoparathyroidism
PTH	Parathyroid hormone
HDR	Hypoparathyroidism–Sensorineural Deafness–Renal Dysplasia syndrome
HRDS/Sanjad-Sakati	Hypoparathyroidism–Retardation–Dysmorphism syndrome
KCS	Kenny–Caffey syndrome
CaSR	Calcium-sensing receptor
ADH1	Autosomal dominant hypocalcemia type 1
APS-1	Autoimmune Polyglandular Syndrome type 1
rhPTH	Recombinant human parathyroid hormone
SR-WGS	Short-read whole genome sequencing
SNV	Single Nucleotide Variant
CNV	Copy Number Variation
SV	Structural Variation
STR	Short Tandem Repeat
MEI	Mobile Element Insertion
OMIM	Online Mendelian Inheritance in Man
NICU	Neonatal Intensive Care Unit
FDA	Food and Drug Administration
EMA	European Medicines Agency
